# Developmental Differences in Filtering Auditory and Visual Distractors During Visual Selective Attention

**DOI:** 10.3389/fpsyg.2018.02564

**Published:** 2018-12-17

**Authors:** Christopher W. Robinson, Andrew M. Hawthorn, Arisha N. Rahman

**Affiliations:** Department of Psychology, The Ohio State University Newark, Newark, OH, United States

**Keywords:** selective attention, cross-modal processing, modality dominance, aging, auditory processing, visual processing

## Abstract

The current experiment examined changes in visual selective attention in young children, older children, young adults, and older adults while participants were instructed to ignore auditory and visual distractors. The aims of the study were to: (a) determine if the Perceptual Load Hypothesis (PLH) (distraction greater under low perceptual load) could predict which irrelevant stimuli would disrupt visual selective attention, and (b) if auditory to visual shifts found in modality dominance research could be extended to selective attention tasks. Overall, distractibility decreased with age, with incompatible distractors having larger costs in young and older children than adults. In regard to accuracy, visual distractibility did not differ across age nor load, whereas, auditory interference was more pronounced early in development and correlated with age. Auditory and visual distractors also slowed down responses in young and older children more than adults. Finally, the PLH did not predict performance. Rather, children often showed the opposite pattern, with visual distractors having a greater cost in the high load condition (older children) and auditory distractors having a greater cost in the high load condition (young children). These findings are consistent with research examining the development of modality dominance and shed light on changes in multisensory processing and selective attention across the lifespan.

## Introduction

Many situations require individuals to focus on a task and ignore irrelevant distractors. For example, children may struggle in school because they have difficulty filtering out irrelevant stimuli and are easily distracted by lively classroom settings (Fisher et al., 2014). Selective attention changes considerably across development (Enns and Akhtar, 1989; Maylor and Lavie, 1998; Huang-Pollock et al., 2002; see also Lane and Pearson, 1982; Plude et al., 1994; Hanania and Smith, 2010, for reviews) and appears to be tied to the slow protracted development of working memory (Diamond, 2006). At the same time, lifespan studies suggest that there may be a U-shaped curve across development, with older adults also being more distracted by irrelevant stimuli than young adults (McDowd and Filion, 1991; Maylor and Lavie, 1998; Alain and Woods, 1999; West and Alain, 2000; Gazzaley et al., 2005; Andres et al., 2006; Poliakoff et al., 2006; Lustig et al., 2007; Yang and Hasher, 2007; Guerreiro et al., 2010; Jost et al., 2011; but see Bell and Buchner, 2007, for findings and review of studies showing no age related increase in distractibility).

### Perceptual Load Hypothesis

Within the visual modality, the Perceptual Load Hypothesis (PLH) predicts which visual distractors interfere with processing (Lavie and Tsal, 1994; Lavie, 1995). Perceptual load often refers to the amount of information presented to participants (e.g., high perceptual load is associated with more information to process), and the PLH predicts that visual distractors will result in greater costs under low perceptual load conditions, due to more available attentional resources to detect the irrelevant information. For example, in Lavie (1995), participants had to quickly respond to targets and ignore distractors. Distractors were either compatible (target and distractor were identical and associated with same response), incompatible (target was associated with one response and distractor was associated with a different response), or neutral (distractors were not associated with any response). Under the high attentional load condition in Experiment 1, participants had to search through six stimuli to identify and respond to a target item. Under low load, set size was one and participants were only presented with a single target and a distractor. The main finding from Lavie (1995), as well as follow up studies, is that incompatible distractors have a greater cost in low load conditions (see also Murphy et al., 2016, for a review).

Studies examining the development of selective attention show that age interacts with perceptual load, which may suggest different developmental timing of early and late filters (Huang-Pollock et al., 2002). For example, children in Huang-Pollock et al. (2002) had more difficulty filtering out irrelevant distractors than adults, but only in the low load condition. Older adults also appear to have more difficulty filtering distractors than young adults, and like children, this effect was most pronounced under the low load condition (Maylor and Lavie, 1998).

Although the PLH provides insight into the relationship between processing demands and distractibility in the visual modality, less research has examined if the PLH also predicts distractibility in the auditory modality or when presented with cross-modal stimuli (Tellinghuisen and Nowak, 2003; Macdonald and Lavie, 2011; Matusz et al., 2015; Broadbent et al., 2018). There is some support that PLH can predict distractibility across sensory modalities. For example, Macdonald and Lavie (2011) manipulated perceptual load in the visual modality and tested detection of sounds in the auditory modality. Consistent with PLH, participants were more likely to detect the tone under low load. However, not all cross-modal selective attention studies support PLH. For example, Tellinghuisen and Nowak (2003) found that auditory distractors were more distracting under high load, which is inconsistent with PLH (see also Murphy et al., 2016, 2017 for reviews of auditory selective attention). Thus, it is unclear if PLH can predict auditory distractibility and distractibility across sensory modalities, and there are also reasons to suspect that these effects may change across development.

### Development of Cross-Modal Processing Throughout the Lifespan

Presenting information to one sensory modality can sometimes dominate or interfere with processing in a second modality (modality dominance). While modality dominance effects are flexible and vary as a function of stimulus familiarity (Sloutsky and Napolitano, 2003; Robinson and Sloutsky, 2004), response demands (Robinson et al., 2016), nature of the task (Welch and Warren, 1980), and signal strength (Alias and Burr, 2004), studies that have employed the same task and stimuli across development often find developmental changes in modality dominance. Early in development, there are numerous situations where the auditory modality dominates visual processing (Lewkowicz, 1988a,b; Sloutsky and Napolitano, 2003; Napolitano and Sloutsky, 2004; Robinson and Sloutsky, 2004, 2010b, 2019; Sloutsky and Robinson, 2008; Nava and Pavani, 2013; see also Robinson and Sloutsky, 2010a for a review), whereas, visual dominance is more likely to be found in adults (Colavita, 1974; Koppen et al., 2008; Sinnett et al., 2008; Ngo et al., 2010, 2011; Nava and Pavani, 2013; see also Sinnett et al., 2007; Spence et al., 2012 for reviews).

A closer examination of modality dominance shows that these effects appear to be changing in childhood, with 6- to 7-year-olds showing evidence of auditory dominance and 9- to 10-year-olds showing adult-like visual dominance (Nava and Pavani, 2013). Auditory to visual shifts across development can also be seen using a variety of tasks such as implicit categorization (Broadbent et al., 2018), Sound Induced Flash illusion (Nava and Pavani, 2013), Colavita visual dominance (Nava and Pavani, 2013; see also Hirst et al., 2018a, for a review), and the Mcgurk Effect (McGurk and MacDonald, 1976; Massaro, 1984; Ross et al., 2011; Hirst et al., 2018b). In all of these studies, effects of auditory input either decrease with age and/or effects of visual input increase with age.

To account for auditory dominance effects in young children, Robinson and Sloutsky (2010a) suggested that sensory modalities are competing for attentional resources. While various accounts have speculated whether sensory modalities share attentional resources or have their own dedicated resources (Wickens, 1984; Spence and Driver, 1996, 1997; Duncan et al., 1997; Eimer and Driver, 2000; Eimer and van Velzen, 2002; Pavani et al., 2004; Sinnett et al., 2007; Macdonald and Lavie, 2011), the current account differs by positing that the auditory modality should win this competition, especially early in development. First, auditory stimuli are almost always dynamic and transient in nature, whereas, visual stimuli are more likely to be presented for prolonged periods of time. Thus, it may be adaptive to first allocate attention to the auditory information before it disappears. Second, given the relative early maturation of the auditory system relative to the visual system (Birnholz and Benaceraff, 1983; see also Jusczyk, 1998, for a review), it is possible that young children might prioritize auditory processing because the sensory system is more developed and provides a stronger signal.

However, it is important to note that not all multisensory contexts attenuate processing and result in modality dominance. Rather, in some situations, multisensory contexts facilitate processing and/or responding. For example, when multisensory stimuli are presented in close spatial or temporal proximity, these stimuli are more likely to be bound into a single percept, and responses to these stimuli are often faster than responses to unimodal stimuli (Miller, 1982; Giard and Peronnet, 1999; Fort et al., 2002; Colonius and Diederich, 2006; Laurienti et al., 2006; Sinnett et al., 2008; Barutchu et al., 2010; Downing et al., 2015). Semantic congruency (e.g., seeing a dog and hearing a dog bark) can also result in enhancements even in the presence of incompatible auditory, visual, and audiovisual distractors. However, under such distracting conditions, multisensory enhancements are lower, with children and adolescence showing greater sensitivity to unisensory and multisensory distractors than adults (Downing et al., 2015).

While there is some evidence of multisensory integration early in infancy (Bahrick et al., 2004, for a review), multisensory integration appears to have a long, protracted development (Gori et al., 2008, 2012; Nardini et al., 2008; Barutchu et al., 2009, 2010; Brandwein et al., 2011; Ross et al., 2011), which might be associated with protracted development of attentional networks (Talsma et al., 2010). For example, while adolescents and adults often respond faster to redundant multisensory cues and violate the race model (Miller, 1982), young children’s responses times are often consistent with the race model, suggesting that they are not optimally binding multisensory information (Barutchu et al., 2010; Downing et al., 2015).

Finally, while a considerable amount of research has examined modality dominance, multisensory integration, and filtering of auditory and visual distractors early in development, less is known about these processes in older adults. It is well established that attention/executive function (Royall et al., 2004) and sensory systems (Corso, 1971; Pitts, 1982; He et al., 1998; Schneider et al., 2000; Liu and Yan, 2007) undergo substantial changes in late adulthood (see also Birren and Schaie, 2006, for a review). Thus, it is possible that cross-modal effects increase into late adulthood, either because of poor filtering of irrelevant cross-modal stimuli (Raz, 2000; Andres et al., 2006; Poliakoff et al., 2006; Lustig et al., 2007; but see Bell and Buchner, 2007, where there were no differences in auditory distraction in young and older adults) or from inverse effectiveness (Meredith and Stein, 1983), with multisensory integration increasing in older adults to compensate for declines in unimodal processing (DeLoss et al., 2013; but see Barutchu et al., 2010, where decreasing the signal to noise ratio in younger participants decreased, multisensory integration). At the same time, other studies show reliance on visual input increases into late adulthood (Thompson and Malloy, 2004; Mahoney et al., 2012; Diaconescu et al., 2013; Sekiyama et al., 2014; Van Gerven and Guerreiro, 2016; Barnhart et al., 2018; Parker and Robinson, 2018; see also Costello and Bloesch, 2017, for a review), which might indicate that visual dominance effects continue to strengthen into late adulthood.

### Goals of Current Study

The primary goal of the current study was to investigate the role of perceptual load and age-related differences in visual selective attention, and whether these effects depend on sensory modality. Participants in the current study were presented with a variation of a flanker task (Eriksen and Eriksen, 1974; Lavie, 1995) where they had to respond to visual targets (bird or dog) and ignore auditory or visual distractors. Participants responded by quickly pressing one button if they saw a dog and by pressing a different button when they saw a bird. We manipulated perceptual load (set sizes of 1 or 6), compatibility of distractors (compatible or incompatible), and modality of distractors (auditory or visual).

Based on research examining the development of selective attention across the lifespan, a U-shaped curve was predicted when examining effects of visual distractors on attention, and these effects should be most pronounced under low load (Maylor and Lavie, 1998; Huang-Pollock et al., 2002). Based on multisensory processing research, interference from auditory distractors should be more pronounced early in development and decrease with age (Sloutsky and Napolitano, 2003; Robinson and Sloutsky, 2004; Nava and Pavani, 2013), and based on Downing et al. (2015), older children should show greater sensitivity to visual distractors than auditory distractors, and a greater sensitivity than adults to all distractors than adults. Finally, it is possible that effects of visual distractors will be stronger in adults and continue to increase into late adulthood (Thompson and Malloy, 2004; Mahoney et al., 2012; Diaconescu et al., 2013; Sekiyama et al., 2014; Barnhart et al., 2018; Parker and Robinson, 2018).

## Materials and Methods

### Participants

We tested 34 children between the ages of 3.6–11.6 years and we used a median split to categorize children into young children and older children. The final sample consisted of 17 young children (7 Females, *M* = 4.81 years, *SD* = 0.73, range 3.59–5.99 years), 17 older children (11 Females, *M* = 8.75 years, *SD* = 1.84, range 6.32–11.55 years), 24 young adults (10 Females, *M* = 19.20 years, *SD* = 2.35, range 18.20–30.00 years), and 16 older adults (10 Females, *M* = 74.93 years, *SD* = 9.33, range 62.28–93.12 years). Children were either tested in a quiet room at their daycare or in a quiet room in the psychology laboratory at The Ohio State University at Newark. The young adults consisted of Introduction to Psychology students from The Ohio State University at Newark and they participated for course credit. Young adults were tested in a quiet room in the psychology laboratory at The Ohio State University at Newark.

Approximately half of the older adults were tested at OSU Newark and the remaining older adults were tested in a quiet room at a Continuing Care Retirement Center (CCRC). Participants tested in the lab were recruited through word of mouth whereas participants tested at the CRCC location were recruited through an existing partnership between the CCRC and The Ohio State University. Parents commuting to campus and older adults received a $10 gift card for their participation. The only criterion for participation was that participants had both hearing and vision that was considered normal or corrected to normal (self-reported). An additional four participants were tested but not included in the final sample due to a failure to complete the experiment (two children), developmental delay (one child), and because of hearing loss (one older adult).

Recruitment and experimental procedures were carried out in accordance with the guidelines and approval of The Ohio State University’s Behavioral and Social Sciences Institutional Review Board, Protocol# 2014B0022, Cross-modal processing across the lifespan. After participants/legal guardians were informed about the nature of the study, adults completed an IRB approved informed consent form. All children in the study verbally assented to participate and guardians also filled out an IRB approved parental consent form.

### Stimuli

**FIGURE 1:**
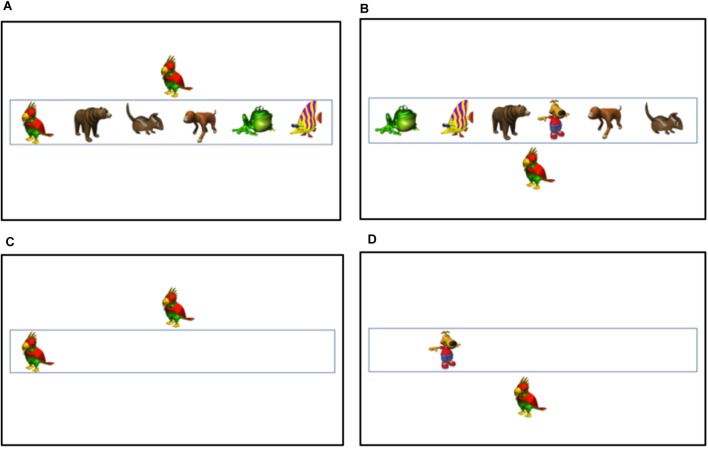
Example trials from the visual distractor conditions: **(A)** High Load Compatible, **(B)** High Load Incompatible, **(C)** Low Load Compatible, and **(D)** Low Load Incompatible.

The visual stimuli consisted of six colorful cartoon animals. Each visual stimulus was presented on a 22″ monitor at approximately 3.80 cm × 5.70 cm, with an approximate horizontal visual angle of 3.63° and vertical visual angle of 5.44°. Two of the visual stimuli were chosen as the targets (Bird and Dog). On each trial, there was only one target, which was either the bird or dog and appeared inside the box. There were also five non-target items. These items also appeared within the box, but stimuli were not associated with a response. For example, in the high load condition, there was one target (either a bird or a dog) and five non-targets (fish, frog, etc.). In the low load condition, there was only one stimulus in the box, and it was the target. Targets and non-targets could appear in any of the six locations. There were also auditory and visual distractors. Visual distractors (bird or dog) were presented centrally above the box or centrally below the box, and participants were instructed to ignore the distractors and only report if there was a bird or dog inside the box. Half of the distractors were compatible (e.g., bird inside the box and bird outside the box) and half were incompatible (e.g., bird inside the box and dog outside the box). See Figures 1A,B for examples of high load visual distractor trials and see Figures 1C,D for examples of low load visual distractor trials.

The auditory distractors consisted of a short bird chirp or dog bark. The animal sounds were downloaded from Marcell et al. (2000), shortened to 800 ms using Audacity software, and saved as 44.1 kHz wav files. Auditory stimuli were presented via headphones at approximately 65–68 dB and were presented at the onset of the visual stimulus. Auditory stimuli were presented to both ears at equal intensity. Auditory distractor trials were identical to visual distractor trials, but the bird and dog visual distractors were removed and replaced by a short sound clip of one of the animal sounds.

### Procedure

Participants in this study sat approximately 60 cm from a computer monitor. They were instructed that a box of animals would appear on the computer screen and that they had to quickly find a bird or a dog inside the box. At this time, a box of animals appeared on the screen and the experimenter pointed to the bird on the screen and then indicated which button to push if they saw the bird. The experimenter also asked the children to push the button associated with the bird. These trials also served as practice trials to make sure they understood the procedure (pay attention to animals inside the box). The computer then presented a picture of the dog and the experimenter pointed to the dog and indicated which button to push if they saw the dog. The experimenter also asked children to push the button associated with the dog. A colorful picture of the bird and dog were also placed next to each button to help children remember the button-animal pairing. Participants were also told that the bird or dog could appear anywhere inside of the box, the bird and the dog would never both be presented in the box at the same time, and that they should respond as fast as and as accurately as possible. Participants were also instructed that the computer would attempt to trick them by presenting birds and dogs above or below the box (visual distractor conditions) or by presenting bird chirps and dog barks through the headphones (auditory distraction conditions). They were instructed to ignore the distractors and only focus on the animals that appear inside the box.

Before beginning the study, the experimenter presented an incompatible trial and checked to make sure participants understood the game. For example, the experimenter might say, “Look, there is a bird inside the box, but there is a dog outside the box. Which button would you push?” The experimenter did not present an actual trial until the participant pressed or said the correct button and s/he understood the game (e.g., pay attention to animals in the box and ignore animals outside of the box). The experimenter started the experiment by pressing the enter key on the keyboard; at this point, the computer randomly started one of the four blocks (high load visual distractors, high load auditory distractors, low load visual distractors, or low load auditory distractors). The four blocks were manipulated within subjects and each block had 48 trials (192 total trials), and trials within each block and block order were randomized for each participant. The target (bird/dog), compatibility of distractors (compatible/incompatible), location of visual distractor (above/below box), and modality of distractor (auditory/visual) were fully counterbalanced within each participant. In the visual distractor condition, the visual targets (bird or dog inside box), non-targets (other animals inside box), and distractors (bird or dog outside box) were presented until the participant made a response. In the two auditory distractor blocks, the visual targets and non-targets were presented until participants made a response, however, the auditory distractors were only presented at the onset of each trial (first 800 ms of the trial). Participants responded to the bird and dog by pressing two USB buttons, and left-right button location was counterbalanced across participants. The whole experiment took approximately 15 min, and response times and accuracies were collected on each trial.

Eighteen of the children (11 Females, *M* = 5.23 years, *SD* = 1.26, range 3.60–7.59 years) reported in the final sample also participated in two additional control conditions where there were no auditory or visual distractors (i.e., high load condition with no distractors and low load condition with no distractors). For these children, there were six different blocks and each block contained 48 trials (288 total trials). These children also completed the four main testing blocks, so their data were included with the full sample, however, we also present their data adjusted to the no distractor baselines at the end of the results section. We were also concerned that the children would be off task, especially since the control conditions significantly increased the duration of the experiment, so we made one additional change to the procedure for this subset of children. The experimenter started each trial when children were on task and looking at the screen, as opposed to trials starting automatically after responding on the previous trial.

## Results

On each trial, participants responded by pressing either the bird or dog button. We coded each correct response as 1 and each incorrect response as 0. See the left side of Table 1 for the mean/median proportion of correct responses and standard deviations broken up by Age, Modality, Load, and Compatibility. We were primarily interested in developmental changes in visual selective attention when participants were instructed to ignore visual distractors and in developmental changes in selective attention when presented with auditory distractors. We begin by focusing on accuracy and response times when participants were presented with visual distractors.

**Table 1:** Mean/Median proportion correct, Mean/Median response times in ms, and (Standard Deviations) across age, distractor modality, and load.

Age/Condition	Compatible Accuracy	Incompatible Accuracy	Compatible reaction times	Incompatible reaction times
**Young Children**				
Visual high	0.92/0.96 (0.11)	0.89/0.96 (0.13)	2379/2095 (1242)	2537/2395 (1169)
Visual low	0.91/0.96 (0.14)	0.90/0.92 (0.11)	1679/1612 (708)	1987/1881 (810)
Auditory high	0.88/0.96 (0.13)	0.69/0.79 (0.30)	1847/1562 (729)	2197/1739 (1038)
Auditory low	0.91/0.96 (0.15)	0.83/0.92 (0.22)	1369/1211 (453)	1768/1380 (1031)
**Older Children**				
Visual high	0.97/1.0 (0.06)	0.95/0.96 (0.04)	2179/1829 (1264)	2635/2098 (1646)
Visual low	0.95/0.96 (0.07)	0.96/0.96 (0.04)	2008/1429 (1919)	2069/1484 (1659)
Auditory high	0.98/1.0 (0.03)	0.98/10.0 (0.03)	2036/1569 (1177)	2287/1601 (1723)
Auditory low	0.96/1.0 (0.06)	0.97/1.0 (0.05)	1610/1356 (744)	1788/1470 (821)
**Young Adults**				
Visual high	0.99/1.0 (0.02)	0.98/1.0 (0.03)	730/732 (106)	760/743 (131)
Visual low	0.97/1.0 (0.05)	0.98/1.0 (0.04)	593/593 (84)	636/641 (98)
Auditory high	0.99/1.0 (0.03)	0.98/0.98 (0.03)	738/707 (161)	753/719 (150)
Auditory low	0.99/1.0 (0.03)	0.98/1.0 (0.02)	589/563 (104)	610/588 (119)
**Older Adults**				
Visual high	0.99/1.0 (0.02)	1.0/1.0 (0.00)	1030/978 (242)	1033/1031 (172)
Visual low	1.0/1.0 (0.01)	1.0/1.0 (0.01)	769/746 (116)	809/846 (110)
Auditory high	1.0/1.0 (0.01)	0.97/0.98 (0.03)	1008/990 (235)	1046/974 (309)
Auditory low	0.99/1.0 (0.01)	0.99/1.0 (0.04)	743/736 (124)	745/747 (27)


### Effects of Visual Distractors

**FIGURE 2:**
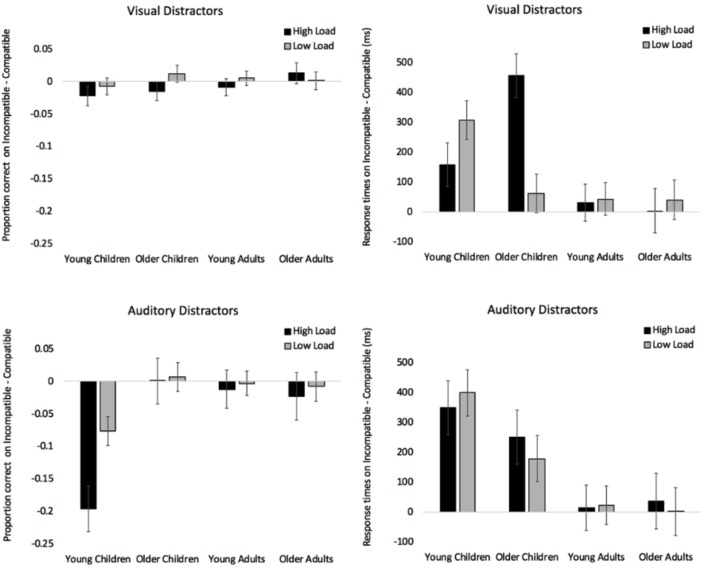
Proportion of correct responses on incompatible trials – compatible trials when presented with visual distractors (top left) and auditory distractors (bottom left). Response times on incompatible trials – compatible trials when presented with visual distractors (top right) and auditory distractors (bottom right). Error bars denote Standard Errors.

To determine if visual compatible and incompatible distractors had different effects on accuracy, we calculated a difference score for each participant (proportion correct on incompatible trials – proportion correct on compatible trials). Difference scores below zero indicate that accuracy on incompatible trials was lower than on compatible trials. Difference scores in the visual distractor conditions were submitted to a 4 (Age: Young Children, Older Children, Young Adults, Older Adults) × 2 (Load: High, Low) mixed-factors ANOVA, with load manipulated within subjects. The analysis revealed no significant effects or interactions. See top-left panel of Figure 2 for difference scores across age and load and see top section of Table 2 for all analyses.

**Table 2:** Age × Load mixed-factors ANOVA examining effects of visual distractors on accuracy/response times (top) and effects of auditory distractors on accuracy/response times (bottom).

Dependent variable effects/Interaction	ANOVA statistics	η*_p_*^2^	Observed power	Non-parametric statistics
**Visual Distractors**				
**Accuracy**				
Load	*F*(1, 70) = 1.02, *p* = 0.316	0.014	0.169	*Z* = -0.53, *p* = 0.593
Age	*F*(3, 70) = 0.92, *p* = 0.435	0.038	0.242	*X*^2^ = 2.92, *p* = 0.404
Load × Age	*F*(3, 70) = 0.58, *p* = 0.628	0.024	0.165	–
Age: High load	*F*(3, 70) = 0.97, *p* = 0.271	0.040	0.253	*X*^2^ = 4.59, *p* = 0.204
Age: Low load	*F*(3, 70) = 0.40, *p* = 0.753	0.017	0.126	*X*^2^ = 4.71, *p* = 0.194
**Response times**				
Load	*F*(1, 70) = 0.95, *p* = 0.334	0.013	0.160	–
Age^∗∗^	*F*(3, 70) = 8.00, *p* < 0.001	0.255	0.987	–
Load × Age^∗∗^	*F*(3, 70) = 5.36, *p* = 0.002	0.187	0.921	–
**Auditory Distractors**				
**Accuracy**				
Load^∗∗^	*F*(1, 70) = 8.05, *p* = 0.006	0.103	0.799	*Z* = -2.78, *p* = 0.005
Age^∗∗^	*F* (3, 70) = 6.52, *p* = 0.001	0.218	0.964	*X*^2^ = 22.01, *p* < 0.001
Load × Age^∗∗^	*F*(3, 70) = 4.10, *p* = 0.010	0.150	0.828	–
Age: High load^∗∗^	*F* (3, 70) = 7.26, *p* < 0.001	0.237	0.979	*X*^2^ = 13.22, *p* = 0.004
Age: Low load^∗^	*F*(3, 70) = 2.91, *p* = 0.040	0.111	0.670	*X*^2^ = 8.78, *p* = 0.032
**Response times**				
Load	*F*(1, 70) = 0.05, *p* = 0.828	0.001	0.055	–
Age^∗∗^	*F*(3, 70) = 9.64, *p* < 0.001	0.292	0.996	–
Load × Age	*F*(3, 70) = 0.19, *p* = 0.906	0.008	0.083	–


*Wilcoxon Signed Ranked test (Z, non-parametric two related samples) and Kruskal–Wallis (X^*2*^, non-parametric independent samples) are also reported. ^∗^p < 0.05 and ^∗∗^p < 0.01.*

It is important to note that the proportion of correct responses across some of the conditions was at, or approached, ceiling. Given concerns of normality, we also analyzed difference scores by using non-parametric analyses. Wilcoxon Signed Ranked Test (effect of load), Kruskal-Wallis (effect of age), and two Kruskal-Wallis tests examining effects of age separately under high and low load (age × load interaction) corroborated the ANOVA. As can be seen on the top-right side of Table 2, none of the non-parametric tests approached significance.

We also examined response times. On each trial, we recorded a response time (timestamp of response – timestamp of stimulus onset). Response times greater than three standard deviations were removed from the analyses, and we only averaged across response times when participants made a correct response. See the right side of Table 1 for mean/median response times and standard deviations broken down by Age, Modality, Load, and Compatibility. As with the accuracy analyses, we calculated difference scores (Incompatible RT – Compatible RT). Values greater than zero indicate that response times on incompatible distractor trials were slower than compatible distractor trials.

Difference scores in the visual distractor conditions were submitted to a 4 (Age: Young Children, Older Children, Young Adults, Older Adults) × 2 (Load: High, Low) mixed-factors ANOVA with load manipulated within subjects. See top-right panel of Figure 2 for difference scores across age and load, and see Table 2 for all analyses. There was a significant effect of Age, and pairwise comparisons with Bonferroni adjustments revealed that young children (*M* = 233 ms, *SE* = 46) were more distracted by incompatible visual distractors than young adults (*M* = 37 ms, *SE* = 39), *p* = 0.010, and older adults (*M* = 22 ms, *SE* = 47), *p* = 0.012. Older children (*M* = 259 ms, *SE* = 46) were also more distracted by the incompatible distractors than young adults, *p* = 0.004, and older adults, *p* = 0.004. Young and older children did not differ, *p* > 0.99. Note that all pairwise comparisons throughout the manuscript were corrected with Bonferroni adjustment.

The analysis also revealed a significant Age × Load interaction (see top-right section of Figure 2 for difference scores). Under high load, simple effects with Bonferroni adjustment revealed that older children significantly differed from young children, *p* = 0.030, young adults, *p* < 0.001, and older adults, *p* < 0.001. There were no differences between young children, young adults, and older adults (*p*’s > 0.875). All simple effects analyses reported in the manuscript were Bonferroni corrected for multiple comparisons. Under low load, tests of simple effects revealed that young children differed from young adults, *p* = 0.016, and older adults, *p* = 0.033. The difference between young and older children did not reach significance, *p* = 0.054, and there were no differences between older children, young adults, and older adults, *p*’s > 0.99. Finally, it is also worth noting that there was only one age group where performance differed under low and high load. Simple effects by load show that response times in older children differed between low and high load, with incompatible visual distractors having a greater cost under high load, *p* < 0.001. The direction in young children was consistent with PLH, but the difference did not reach significance, *p* = 0.150.

### Effects of Auditory Distractors

To determine if compatible and incompatible auditory distractors had different effects on accuracy across development, we submitted difference scores (proportion correct on incompatible trials – proportion correct on compatible trials) in the auditory distractor conditions to a 4 (Age: Young Children, Older Children, Young Adults, Older Adults) × 2 (Load: High, Low) mixed-factors ANOVA, with load manipulated within subjects. The analysis revealed main effects of Age and Load, and the Age × Load interaction was also significant (see bottom-left panel of Figure 2 for difference scores and bottom of Table 2 for ANOVA analyses). Pairwise comparisons examining the effect of age revealed that young children (*M* = -0.136, *SE* = 0.03) differed from older children (*M* = 0.004, *SE* = 0.03), *p* = 0.002, young adults (*M* = -0.008, *SE* = 0.02), *p* = 0.002, and older adults (*M* = -0.016, *SE* = 0.03), *p* = 0.011. None of the other age groups differed from each other, *p*’s > 0.99. The effect of load was driven by incompatible auditory distractors having a greater cost on accuracy in the high load condition (*M* = -0.058, *SE* = 0.02) than in the low load condition (*M* = -0.020, *SE* = 0.01). As can be seen in the bottom right section of Table 2, the Wilcoxon Signed Ranked test and Kruskal-Wallis analyses corroborated the results from the ANOVA, and a series of Mann-Whitney U tests confirmed that the youngest age group differed from the other three age groups, *p*’s < 0.004.

Simple effects were conducted to break down the Age × Load interaction (see Figure 2). Under high load, young children significantly differed from older children, *p* = 0.001, young adults, *p* = 0.001, and older adults, *p* = 0.006, and Mann-Whitney U tests confirmed that all three age groups differed from each other, *p*’s < 0.026. There were no differences between older children, young adults, and older adults, *p*’s > 0.99. Under low load, young children did not differ from older children, *p* = 0.064, however, this effect reached significance when using a Mann-Whitney *U*-test, *p* = 0.015, which does not assume normality. None of the other comparisons were significant using an alpha level of 0.05, *p*’s > 0.095. Finally, it is important to note that young children were the only group to show a significant difference between the low and high load conditions, with incompatible auditory distractors having a greater cost under high load, *p* < 0.001. Load did not have an effect in any of the other age groups, *p’*s > 0.584.

To determine how auditory distractors affected the speed of responding, we submitted difference scores (Incompatible RT – Compatible RT) in the auditory distractor conditions to a 4 (Age: Young Children, Older Children, Young Adults, Older Adults) ×2 (Load: High, Low) mixed-factors ANOVA with load manipulated within subjects. See bottom-right panel of Figure 2 for difference scores and see bottom of Table 2 for all analyses. The ANOVA only revealed an effect of Age, and pairwise comparisons revealed that younger children (*M* = 375 ms, *SE* = 57) significantly differed from young adults (*M* = 18 ms, *SE* = 48), *p* = 0.002, and older adults (*M* = 20 ms, *SE* = 59), *p* = 0.002. The difference between older children (*M* = 211 ms, *SE* = 0.57) and young adults did not reach significance, *p* = 0.073, and none of the other comparisons were significant, *p’s* > 0.139.

### Correlations Between Age, Accuracy, and Response Times

Given previously reported auditory to visual dominance shifts that occur between 6- and 10-years of age (e.g., Nava and Pavani, 2013), we further examined the relationship between age, visual distractibility, and auditory distractibility by examining correlations across the young and older children (*N* = 34, range 3.6–11.6 years). The correlations between age, accuracy (difference scores), and response times (difference scores) across the different load and modality manipulations are presented in Table 3. Due to concerns about normality with the accuracy data, we used Spearman rho to examine relationships between age and distractibility. As can be seen in the table, age in years only correlated with accuracy when presented with auditory distractors. More specifically, age was positively correlated with difference accuracy scores when presented auditory distractors under high load, *p* < 0.001, and low load, *p* = 0.019. Recall that higher accuracy scores denote less distractibility. The correlation between age and accuracy in the visual distractor condition (low load) did not reach significance, *p* = 102, however, the positive correlation suggests that visual distractibility might be decreasing rather than increasing with age. Finally, there was some evidence that auditory and visual accuracy were correlated under low load, *p* = 0.051.

**Table 3:** Spearman rho correlations between age, accuracy (difference scores), and response times (difference scores) across load and modality of distractor.

	Age	High load auditory accuracy [HA Acc]	Low load auditory accuracy [LA Acc]	High load visual accuracy [HV Acc]	Low load visual accuracy [LV Acc]	High load auditory RTs [HA RT]	Low load auditory RTs [LA RT]	High load visual RTs [HV RT]	Low load visual RTs [LV RT]
Age	-	0.60**	0.40*	-0.01	0.29	-0.23	0.01	0.25	-0.24
HA Acc		-	0.15	-0.13	0.17	-0.01	0.20	0.03	0.11
LA Acc			-	-0.20	0.34	0.07	-0.18	0.23	-0.09
HV Acc				-	-0.26	-0.16	0.13	-0.08	-0.06
LV Acc					-	-0.03	0.18	0.18	0.17
HA RT						-	0.11	-0.14	0.10
LA RT							-	0.06	0.31
HV RT								-	0.01
LV RT									-


*^∗^p < 0.05 and ^∗∗^p < 0.01.*

### No Distractor Control Conditions

One limitation of the current study was that many of the participants only completed four tasks and there were no baseline conditions (i.e., no distractor conditions). Thus, it is unclear if differences between compatible and incompatible distractors stem from compatible distractors facilitating responding, possibly due to multisensory integration, or from incompatible distractors interfering with processing. Eighteen of the children reported in the previous analyses also completed two baseline conditions (i.e., high load no distractors and low load no distractors). If compatible distractors facilitate processing, then performance on compatible trials should exceed the no distractor baseline. However, if differences stem from incompatible distractors interfering with processing, then performance on incompatible trials should be below the no distractor baseline.

#### Effects of Visual Distractors

**FIGURE 3:**
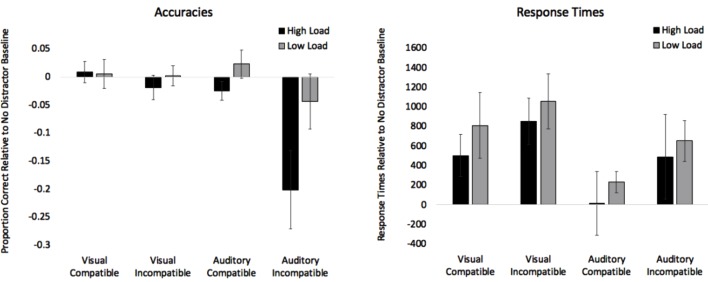
Proportion of correct responses adjusted to the no distractor baselines (compatible – baseline and incompatible – baseline) are presented on the left. Values below 0 indicate interference. Response times adjusted to the no distractor baselines (compatible – baseline and incompatible – baseline) are presented on the right. Values above 0 indicate interference. Error bars denote Standard Errors.

Difference scores for compatible trials were computed by subtracting no distractor trials from compatible trials (i.e., Compatible Accuracy – No Distractor Accuracy), and difference scores for incompatible trials were computed by subtracting no distractor trials from incompatible trials (i.e., Incompatible Accuracy – No Distractor Accuracy). Scores greater than zero indicate facilitation effects and scores below zero indicate interference effects. Accuracy difference scores in the visual distractor conditions were submitted to a 2 (Load: High, Low) × 2 (Compatibility: Compatible, Incompatible) repeated measures ANOVA. The analyses revealed no significant main effects and the Load × Compatibility interaction was not significant. See top panel of Table 4 for analyses and visual trials on the left side of Figure 3 for difference scores.

**Table 4:** Load × Compatibility ANOVA examining effects of visual distractors on accuracy and response times (top), and Load × Compatibility ANOVA examining effects of auditory distractors on accuracy and response times (bottom).

Dependent variable effects/Interaction	ANOVA statistics	η_p_^2^	Observed power
**Visual Distractors**			
**Accuracy**			
Load	*F*(1, 17) = 0.10, *p* = 0.756	0.006	0.060
Compatibility	*F*(1, 17) = 1.33, *p* = 0.264	0.073	0.193
Load × Compatibility	*F*(1, 17) = 0.67, *p* = 0.424	0.038	0.121
**Response Times**			
Load	*F*(1, 17) = 0.45, *p* = 0.512	0.026	0.097
Compatibility^∗∗^	*F*(1, 17) = 13.93, *p* = 0.002	0.450	0.940
Load × Compatibility	*F*(1, 17) = 0.23, *p* = 0.639	0.013	0.074
**Auditory Distractors**			
**Accuracy**			
Load^∗∗^	*F*(1, 17) = 12.05, *p* = 0.003	0.415	0.905
Compatibility^∗^	*F*(1, 17) = 5.64, *p* = 0.030	0.249	0.610
Load × Compatibility	*F*(1, 17) = 4.38, *p* = 0.052	0.205	0.506
**Response times**			
Load	*F*(1, 17) = 0.18, *p* = 0.678	0.010	0.068
Compatibility^∗∗^	*F*(1, 17) = 21.03, *p* < 0.001	0.553	0.991
Load × Compatibility	*F*(1, 17) = 0.48, *p* = 0.828	0.003	0.055


*^∗^p < 0.05 and ^∗∗^p < 0.01.*

Two difference scores were created for response times. Difference scores for compatible trials were computed by subtracting the no distractor baseline from compatible trials (i.e., Compatible RT – No Distractor RT), and difference scores for incompatible trials were computed by subtracting no distractor trials from incompatible trials (i.e., Incompatible RT – No Distractor RT). Difference scores less than zero indicate that distractors sped up responses, and scores above zero indicate that distractors slowed down processing. Response times difference scores in the visual distractor conditions were submitted to a 2 (Load: High, Low) × 2 (Compatibility: Compatible, Incompatible) repeated measures ANOVA. See Table 4 for analyses and visual trials on the right side of Figure 3 for difference scores. The analysis only revealed an effect of Compatibility with response times on incompatible trials (*M* = 955 ms, *SE* = 186) being slower than compatible trials (*M* = 658 ms, *SE* = 183). Both means were above zero, which suggests that there was no evidence of facilitation effects, and the incompatible distractors slowed down responses more than compatible distractors.

#### Effects of Auditory Distractors

As with the visual analyses, we first examined effects of the distractors on accuracy and then we focus on response times. Accuracy difference scores in the auditory distractor conditions were submitted to a 2 (Load: High, Low) × 2 (Compatibility: Compatible, Incompatible) repeated measures ANOVA. See Table 4 for analyses and auditory trials on the left side of Figure 3 for difference scores. The analyses revealed an effect of Load, with the mean in the high load condition (*M* = -0.113, *SE* = 0.04) being significantly lower than the low load condition (*M* = -0.010, *SE* = 0.03). The analyses also revealed an effect of Compatibility, with the incompatible mean (*M* = -0.123, *SE* = 0.05) being significantly lower than the compatible mean (*M* = -0.001, *SE* = 0.01). The Load × Compatibility interaction did not reach significance.

Response time difference scores in the auditory distractor conditions were submitted to a 2 (Load: High, Low) × 2 (Compatibility: Compatible, Incompatible) repeated measures ANOVA. See Table 4 and auditory trials on the right side of Figure 3 for difference scores. The analysis only revealed an effect of Compatibility with response times on incompatible trials (*M* = 571 ms, *SE* = 199) being slower than compatible trials (*M* = 124 ms, *SE* = 165).

## General Discussion

The primary goal of the current study was to investigate the role of perceptual load and age-related changes differences in visual selective attention, and whether these effects depend on sensory modality. To address this goal, we asked participants to quickly search for visual targets (image of bird or dog) inside of a pre-specified area on a computer screen, ignore auditory and visual distractors, and quickly respond if they saw a bird or a dog. We manipulated perceptual load by increasing the number of animals presented on the screen, and we also manipulated the modality of distractors (auditory vs. visual). Half of the distractors were compatible (e.g., target was a bird and distractor was also a bird) and the remaining trials were incompatible (e.g., target was a bird and distractor was a dog). Visual distractors appeared above or below the pre-specified location on the screen and compatible and incompatible auditory distractors were presented via headphones.

The study revealed several important findings regarding auditory and visual distractibility across development (i.e., poorer performance on incompatible trials relative to compatible trials). When examining the effects of visual distractors on accuracy, distractibility did not differ across age nor load. However, visual distractibility did differ across age when examining response times. Young and older children were more distracted than adults. Perceptual load also interacted with age, with older children being more distracted under high load than low load.

Auditory distractors affected both accuracy and response times. When examining the effects of auditory distractors on accuracy, auditory distractors had a larger cost under high load and young children were more distracted than the other age groups. However, perceptual load interacted with age. Young children were more distracted by the auditory distractors under high load. Analyses of response time data also show that young children were more distracted by auditory distractors than the other age groups.

Additional analyses and controls further highlight the nature of these effects. First, we examined the correlation between age, accuracy, and response times across the different conditions in the two youngest age groups. Age only correlated with accuracy in the auditory distractor conditions – auditory distractibility decreased with age. We also compared performance on compatible and incompatible trials to no distractor baseline conditions. There was no evidence that compatible distractors facilitated processing in young children above the no distractor conditions. Rather, differences between compatible and incompatible trials, when found, resulted from incompatible distractors interfering more than compatible distractors.

### Perceptual Load Hypothesis

Based on research examining the development of selective attention across the lifespan, a U-shaped curve was predicted when examining effects of visual distractors on visual selective attention, and these effects were hypothesized to be the most pronounced under low load (Maylor and Lavie, 1998; Huang-Pollock et al., 2002). While response time data show that children were more distracted by incompatible distractors than adults, the pattern of results was inconsistent with PLH (Lavie and Tsal, 1994; Lavie, 1995). For older children, incompatible visual distractors had a greater cost under high load, not low load. In regard to older adults, accuracies approached ceiling for both young and older adults, and when examining response times, there was no evidence that older adults were more distracted than young adults.

While many studies have examined PLH in the visual modality, less research has examined if PLH can predict distractibility across sensory modalities (Tellinghuisen and Nowak, 2003; Macdonald and Lavie, 2011; Matusz et al., 2015; Broadbent et al., 2018). Under some situations, the PLH predicts auditory distractibility (Macdonald and Lavie, 2011), whereas, under other situations, effects of auditory distractors are greatest under high load (Tellinghuisen and Nowak, 2003). There are too many methodological differences across the studies to make strong conclusions, however, it is important to note that Macdonald and Lavie (2011) examined effects of perceptual load on auditory processing, whereas, Tellinghuisen and Nowak (2003) examined effects of perceptual load on visual processing. The current study used a task more similar to Tellinghuisen and Nowak (2003) and found a similar pattern of results. More specifically, when effects of perceptual load were found, these effects stemmed from auditory distractors interfering more under high load.

One interesting finding from the current study is that children’s accuracy and response time data were often inconsistent with PLH (i.e., greater distractibility under high load). It is important to note that this pattern was only found in children and was found for both auditory and visual distractors. One possible explanation for this finding is that this effect stems from poor selective attention and/or slower processing speed in children. However, these explanations cannot account for the pattern of results because it also predicts a greater slow down on compatible trials. It is also possible that the perceptual load manipulations in the current study increased working memory load, especially for young children. While the PLH predicts increased distractibility under low perceptual load, working memory load manipulations sometimes show the opposite pattern, with increased distractibility under high working memory load (Lavie et al., 2004). While speculative, it is possible that children with less top-down control of selective attention were more successful at focusing their attention under low working memory load conditions, whereas, the additional stimuli in the high load condition taxed working memory and made it more difficult for them to focus attention and filter out distractors.

### Development of Cross-Modal Processing Throughout the Lifespan

Some multisensory contexts can result in modality dominance, with one modality attenuating processing in another modality. While modality dominance effects are flexible in nature (Welch and Warren, 1980; Sloutsky and Napolitano, 2003; Alias and Burr, 2004; Robinson and Sloutsky, 2004; Robinson et al., 2016), studies using the same stimuli and procedures across development, often show that young children pay more attention to auditory information, whereas, adults pay more attention to the visual information (e.g., Robinson and Sloutsky, 2004; Nava and Pavani, 2013; see also Robinson and Sloutsky, 2010a, for a review). Moreover, there are reasons to believe that this auditory to visual shift appears between 6 and 10 years of age (Nava and Pavani, 2013). The current study provides some support for this shift across development. In particular, while auditory distractors decreased accuracy and slowed down responses in young children, auditory distractibility decreased with age, and age correlated with accuracy in both the high and low load conditions. While the current study did not directly compare costs of auditory and visual distractibility, the current findings are consistent with Downing et al. (2015). In particular, children showed greater sensitivity to unisensory and multisensory distractors than adults. Moreover, perceptual load manipulations only affected sensitivity to visual distractors in older children, around the same age where Downing et al. (2015) found support for visual dominance. However, it is also worth noting that developmental changes in the current study were not driven by increased interference from the visual modality. Rather, developmental effects stemmed from less cross-modal interference and/or improved filtering of auditory information across development.

Decreased cross-modal interference across childhood raises interesting questions regarding the development of sensory systems and attention. For example, according to early integration accounts (e.g., Gibson, 1966; Werner, 1973; Bower, 1974), sensory modalities are highly interconnected at birth and become more differentiated with age, whereas, according to late integration accounts (e.g., Piaget, 1952; Birch and Lefford, 1963, 1967), sensory modalities are initially independent and become more integrated with age. While there is evidence to support both accounts (see Lewkowicz, 1994, 2000; Robinson and Sloutsky, 2010a, for reviews), the finding that intersensory interactions decreases with age (i.e., weakened auditory interference in the current study) provides additional support for the early integration account.

It is unclear what is driving decreased auditory interference, however, we will speculate on a few mechanisms. First, it is possible that decreased cross-modal interference and/or increased independence stems from intersensory connections being pruned during childhood. Second, and possibly related to intersensory pruning, it is possible that attentional resources are initially shared across modalities (due to many intersensory connections), and with pruning, sensory modalities develop dedicated pools of attentional resources. It is also possible that decreased interference stems from better control of endogenous attention, with adults being more efficient at filtering cross-modal distractors. However, it is important to note that all three of these accounts posit a general increase in sensory independence with age, which is inconsistent with a body of research showing that multisensory integration effects typically increase during childhood and adolescence (Gori et al., 2008; Nardini et al., 2008; Barutchu et al., 2009, 2010; Brandwein et al., 2011; Ross et al., 2011).

### Limitations and Future Directions

While this study sheds light on developmental changes in filtering auditory and visual distractors, there are some limitations. First, there was no evidence that auditory or visual distractors slowed down responses or decreased accuracy in young or older adults. One possible explanation for this finding, or lack of, is that the task was designed for younger children (e.g., longer stimulus presentations, etc.), which would explain why adults’ accuracies approached ceiling. However, it is also important to note that sample sizes within each age group were relatively small with a wide range in age, thus, future research will need to increase sample size and use tighter developmental windows. Second, it is important to note that participants in the current study were only exposed to the auditory stimuli for less than a second, whereas, visual stimuli were presented until a response was made. The relatively short auditory stimulus duration compared to the more protracted visual stimulus might also explain different patterns of findings compared to previous research (Tellinghuisen and Nowak, 2003). More specifically, Tellinghuisen and Nowak (2003) found auditory stimuli affected young adults’ visual responses, whereas, the current study did not find any evidence that auditory distractors affected adults’ responses. However, we did find auditory interference in young children with a decrease across development, which is consistent with modality dominance research. It is also important to note that auditory distractors in the current study were presented at equal levels to both ears, whereas, visual distractors were displaced (i.e., above or below the target area). It is possible that auditory stimuli may have been more likely to capture and interfere with visual processing, whereas, visual distractors that were spatially displaced were less likely to be detected. While this is possible, it appeared to only affect children’s performance.

Additional research will also need to examine how visual distractors affect auditory processing. Recall that previous research has also shown increased reliance on visual information in older adults (Thompson and Malloy, 2004; Diaconescu et al., 2013; Sekiyama et al., 2014; Van Gerven and Guerreiro, 2016; Barnhart et al., 2018; Parker and Robinson, 2018; see also Costello and Bloesch, 2017, for a review), and increased attention to visual information can sometimes come with a cost – slowed or less accurate auditory processing (e.g., Barnhart et al., 2018; Parker and Robinson, 2018). Thus, it is possible that more pronounced effects of aging would have been found if we also examined effects of visual distractors and perceptual load on auditory processing. Finally, it is important to note that we assessed vision and hearing loss via self-report, and proper screening might be needed to fully understand how changes in auditory and visual acuity (some of which might not be reported) affect cross-modal attention and multisensory integration across the lifespan.

## Conclusion

In summary, the current findings highlight changes in selective attention and filtering across the lifespan, with incompatible distractors having a greater cost on processing in children than in young and older adults. While both auditory and visual distractors slowed down children’s responses, only auditory distractors decreased accuracy and auditory distractibility correlated with age. Finally, while there is a considerable amount of support for the PLH in adults, the current study suggests that this hypothesis has difficulty accounting for filtering of auditory and visual distractors in young children.

## Ethics Statement

This study was carried out in accordance with the recommendations of Behavioral and Social Science Institutional Review Board at The Ohio State University, with written informed consent from all subjects. All subjects gave written informed consent in accordance with the Declaration of Helsinki. The protocol (2014B0022) was approved by the Behavioral and Social Science Institutional Review Board at The Ohio State University.

## Author Contributions

CR designed and carried out the study with hypothesis blind research assistants. AH analyzed the data, wrote up the results section, and presented the findings at several conferences. AR wrote up the first draft of the paper (excluding results), collected some of the developmental data, and presented some of the findings at the Midwestern Psychological Association. All authors contributed to manuscript revision, read, and approved the submitted version.

## Conflict of Interest Statement

The authors declare that the research was conducted in the absence of any commercial or financial relationships that could be construed as a potential conflict of interest.
